# A randomized clinical trial on the effects of bupropion and buprenorphine on the reduction of methamphetamine craving

**DOI:** 10.1186/s13063-019-3554-6

**Published:** 2019-07-30

**Authors:** Jamshid Ahmadi, Ali Sahraian, Mehdi Biuseh

**Affiliations:** Substance Abuse Research Center, Shiraz University of Medical Sciences, Chamran BLVD, Hafez Hospital, Shiraz, Iran

**Keywords:** Buprenorphine, Bupropion, Methamphetamine withdrawal craving

## Abstract

**Background:**

The purpose of this study was to compare the effect of 300 mg of bupropion and 8 mg of buprenorphine per day on the treatment of methamphetamine withdrawal cravings over a 2-week treatment interval.

**Method:**

Sixty-five methamphetamine-dependent men who met the DSM-IV-TR (*Diagnostic and Statistical Manual of Mental Disorders*, 4th edition, text revision) criteria for methamphetamine dependence and withdrawal were randomly divided into two groups. Subjects randomly received 300 mg of bupropion or 8 mg of buprenorphine per day in a psychiatric ward. Of the 65 subjects, 35 (53.8%) received buprenorphine and 30 (46.2%) received bupropion. The subjects were assessed by using methamphetamine craving score, interview, and negative urine drug test.

**Findings:**

There were no statistically significant differences between the two groups in regard to age, education, duration of methamphetamine dependency, marital status, employment, and income. The mean ages were 32.8 years (standard deviation (SD) = 7.26, range = 22 to 59) for the buprenorphine group and 32.21 years (SD = 8.45, range = 17 to 51) for the bupropion group. All 65 patients completed the 2-week study. Both medications were effective in the reduction of methamphetamine cravings. Reduction of craving in the buprenorphine group was significantly more than the bupropion group (*P* = 0.011). Overall, a significant main effect of day (*P* <0.001) and group (*P* = 0.011) and a non-significant group-by-day interaction (*P* >0.05) were detected.

**Conclusions:**

The results support the safety and effectiveness of buprenorphine and bupropion in the treatment of methamphetamine withdrawal craving. Administration of 8 mg of buprenorphine per day can be recommended for the treatment of methamphetamine withdrawal cravings. We should note that it is to be expected that craving decreases over time without any medication. So the conclusion may not be that bupropion and buprenorphine both lower the craving. As the buprenorphine is superior to bupropion, only buprenorphine does so for sure.

**Trial registration:**

Iranian Registry of Clinical Trials (IRCT) registration number: IRCT2015010320540N1. Date registered: April 10, 2015.

## Background

Mental disorders have been growing problems around the world [1, 2]. Among psychiatric disorders, substance use disorders and substance-related disorders, involving mainly stimulants such as methamphetamines and cocaine, have been regarded as a developing problem worldwide. Currently, methamphetamine abuse and methamphetamine-induced psychiatric presentations to the hospitals and out-patient centers are growing problems [3–16].

Methamphetamine abuse induces an elevated mood associated with increased wakefulness, physical activity, and energy [7]. Elongated use repeatedly ends to increase drug abuse, decreased weight, increased aggression, increased violence, poor impulse control, long-term health consequences, unstable mood, unstable affect, severe dependency, memory deficits, poor concentration, delusions, and hallucinations [12, 13].

Methamphetamine is abused globally. In the US, 18 million people over age 12 have used methamphetamine during their life [12]. Similar to other addictions, methamphetamine addiction is a chronic relapsing disorder requiring effective pharmacotherapies to help the prevention of relapse.

Previously in Iran, methamphetamine was illegally smuggled in from other regions of the globe, mostly the West [10], but currently it is illegally synthesized and prepared here in “underground” laboratories. It should be mentioned that the methamphetamine illegally synthesized in Iran is much more powerful and is frequently associated with psychosis [10]. A single episode of methamphetamine abuse may induce visual or auditory hallucinations and also persecutory delusions. We have visited and interviewed patients who became psychotic after smoking methamphetamine on only one occasion [10].

Buprenorphine administration is for the treatment of opioid withdrawal symptoms, and bupropion is for the treatment of nicotine dependence [7]. Now we are administering buprenorphine as a new application for the treatment of severe methamphetamine withdrawal cravings because we theorize that (our rationale) the biochemistries involved in methamphetamine and buprenorphine are more or less the same (both medications involve the endogenous opioid system) [7, 9–11].

Buprenorphine, a semi-synthetic partial agonist at mu-opioid receptors and antagonist at delta- and kappa-opioid receptors, has been investigated largely for the treatment of opioid use disorder [7]. It is considered safer than methadone [7, 9], and 8 mg of buprenorphine is as effective as 60 mg of methadone [9]. Buprenorphine is well absorbed after sublingual administration [7]. In animals, buprenorphine shows a flattened or inverted U-shaped curve, and there are dose-correlated rises in anti-nociceptive effect at lower doses and either no greater anti-nociception or a decrease in effect at larger doses [7–11]. Buprenorphine has typical mu-opioid agonist effects, such as sedation, analgesia, and euphoria, and also its partial agonist action at mu-opioid receptors has favored the usage of buprenorphine over methadone; particularly, the minimal respiratory depressant effects of buprenorphine produce greater safety [9–11].

Substances such as alcohol, methamphetamine, and cocaine stimulate release of dopamine from cells originating in the brain’s ventral tegmental area (VTA) region, which is a component of a neuronal circuit called the mesolimbic dopamine system and is connected with behavioral reward and motivation. After exposure to alcohol, methamphetamine, or cocaine, dopamine released into the nucleus accumbens and prefrontal cortex reinforces alcohol-, methamphetamine-, and cocaine-seeking behaviors [17–20].

To the best of our knowledge, based in part on a review of the literature, few studies on this matter (comparing the effects of buprenorphine with those of bupropion for the treatment of severe methamphetamine cravings) have been published [9, 10].

Furthermore, we are administering bupropion as a new approach for the treatment of methamphetamine withdrawal craving because we hypothesize that (our rationale) the biochemistries engaged in methamphetamine and bupropion use are more or less alike (both medications boost the level of dopamine) [7, 9, 11].

This study is a clinical trial that presents data obtained from comparing sublingual buprenorphine with oral bupropion in the treatment of severe methamphetamine withdrawal craving. The primary goal of the present research was to appraise the efficacy of 8 mg of sublingual buprenorphine and 300 mg of oral bupropion per day in the treatment of methamphetamine withdrawal cravings. Craving is a major feature of substance use disorders, including stimulant use disorders, as evidenced by its recent addition to the diagnostic criteria for these disorders in the *Diagnostic and Statistical Manual of Mental Disorders*, 5th edition (DSM-5) (American Psychiatric Association) [7], and it continues even after detoxification to promote relapse [7].

## Methods and Materials

### Subjects

This study was a randomized, double-blind clinical trial of methamphetamine-dependent men who had been referred to the main psychiatric hospital affiliated with the Shiraz University of Medical Sciences. Severe methamphetamine dependence and withdrawal were diagnosed on the basis of the *Diagnostic and Statistical Manual of Mental Disorders*, 4th edition, text revision (DSM-IV-TR) criteria by a board-certified psychiatrist through the Structured Clinical Interview for DSM-IV, clinical version (SCID-I).

In order to have eligibility, subjects were examined and questioned by a board-certified psychiatrist at screening. Prior to each interview, we described the aims of the study, guaranteed confidentiality, and obtained written informed consent. The interviews and examinations were achieved on the premises of the treatment hospital because it appeared to be a non-threatening and suitable environment. Family members, friends, or relatives accompanied subjects to the hospital; this attendance provided a condition to confirm the data and information acquired from the patients.

The inclusion criteria were (1) daily use of methamphetamine for at least 12 months, (2) being male (only men were selected for this study because this main psychiatric ward admits only male patients), and (3) having a positive urine toxicology for methamphetamine.

The exclusion criteria were (1) unstable medical conditions, organic mental disorders, major medical diseases (hepatic, renal, cardiovascular, pulmonary, gastrointestinal, or malignant diseases), or any type of psychosis; (2) intolerable complications arising from the use of buprenorphine/bupropion; and (3) dependency to or abuse of drugs/substances other than methamphetamine (excluding tobacco).

All subjects provided written informed consent before going into the clinical trial. The study was approved and monitored by the ethics committee of Shiraz University of Medical Sciences, which adheres to the Declaration of Helsinki Ethical Principles for Medical Research involving human subjects.

### Randomization

We employed a standard randomization procedure generated by computer to have a random sample set for selecting participants from the patients who met the inclusion criteria and had a desire to participate in the study. In a double-blind manner, the individuals were allocated to groups of either buprenorphine or bupropion by using a random allocation method.

### Procedure

The research team was adequately trained and includes an addiction psychiatrist, general psychiatrist, general physician, psychologist, nurse, and statistician. The pills had the same shape and color. The patients and the research staff were blind to the consuming medications for the period of the study. The ratings and interviews were carried out by a fully trained physician who was unaware of medications and adverse events. All of the pills, including buprenorphine/bupropion, had the same color and shape. During the trial, no other intervention was allowed.

Patients were exactly and precisely monitored and assessed for 14 days. Outcome was measured every day by scoring of craving based on the craving scale, a daily interview for side/adverse effects by a trained physician, and twice-a-week negative urine drug tests (thin layer chromatography, or TLC).

Tolerability and safety of buprenorphine and bupropion were evaluated by daily interview for any side effects, spontaneously reported adverse event data, and rates of premature termination for side effects.

A valid and reliable visual analogue scale that has been used previously [10, 14–16] was used to measure the methamphetamine withdrawal craving; the scale is from 0 to 10 (0 = no craving and 10 = severe desire, craving, or temptation all the time). Patients responded to the prompt, “Rate your craving over the past day”. Measurements of craving were taken each morning. We also instructed the subjects precisely about scoring. In addition, a positive urine drug test for methamphetamine (TLC) at the beginning of the protocol and a negative urine drug test twice a week during the study were required.

Subjects were randomly allocated onto 8 mg/d of sublingual buprenorphine without naloxone (4 mg of buprenorphine twice a day) or 300 mg/d of oral bupropion (150 mg of bupropion twice a day). A trained nurse administered the medications. Subjects received treatment for 14 days.

It should be mentioned that, on the first day, patients who were blind to the medications received only 4 mg of buprenorphine once a day or bupropion 150 mg once a day and, from the second to the 14th day, received 8 mg/d of buprenorphine (4 mg of sublingual buprenorphine twice a day) or 300 mg/d of bupropion (150 mg of oral bupropion twice a day).

### Analysis

Data analysis was performed by using SPSS version 18. Repeated measure two-way analysis of variance (ANOVA) and Student *t* tests analyses were used to test for differences in means, and chi-squared analyses were used to test for differences in frequencies. We used a mixed design “two-way ANOVA”; time (day) was a repeated measure factor, and group (bupropion versus buprenorphine) was the between-subjects factor. Then the crux of the analysis is testing the interaction term (time-by-group) and reporting the associated inferential statistics. All *P* values were two-sided, and statistical significance was set at a 5% level.

## Results

**Fig. 1:**
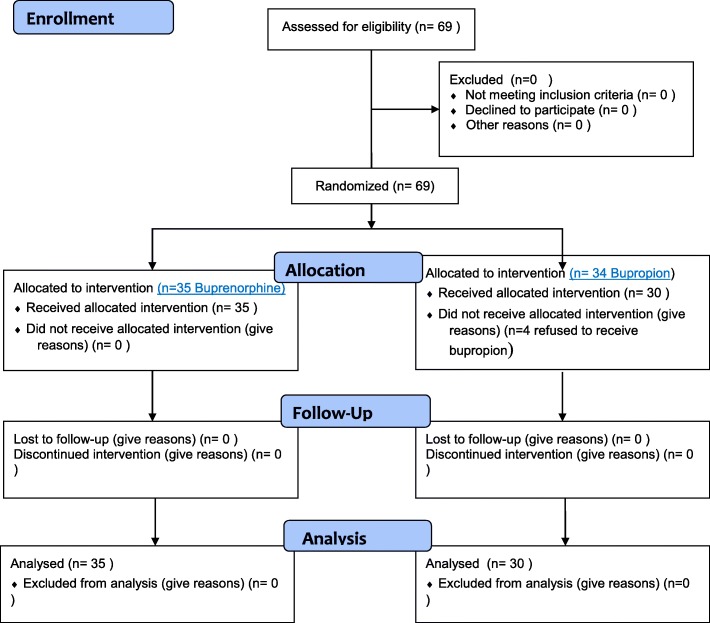
Consolidated Standards of Reporting Trials (CONSORT) flowchart of the patients in this trial

**Fig. 2:**
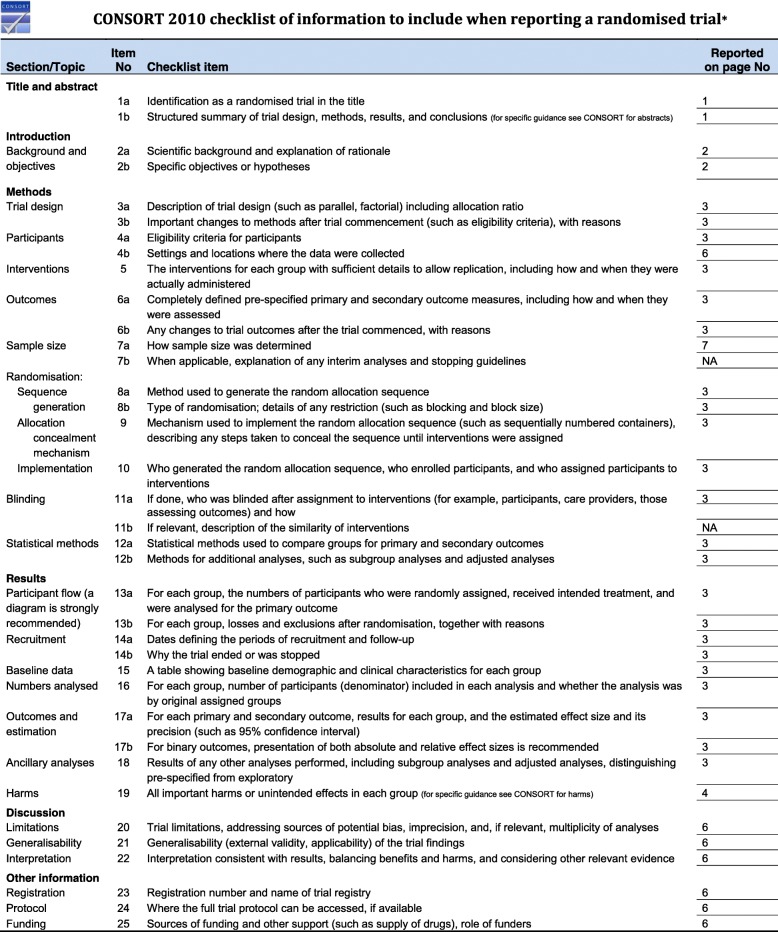
Consolidated Standards of Reporting Trials (CONSORT) 2010 checklist of information to include when reporting a randomized trial

The CONSORT (Consolidated Standards of Reporting Trials) flow and checklist of patients in the trial are shown in Figs. 1 and 2. Seventy-one patients were screened to enter this trial. Of the 69 patients who were randomly allocated to one of the two groups, 34 patients were in the bupropion group and 35 patients were allocated to the buprenorphine group. Four patients of the bupropion group refused to receive the medication at the beginning of the trial.

All 65 subjects completed the 2-week period. Therefore, the data were collected from 65 methamphetamine-dependent men whose mean age was 32.98 years (standard deviation (SD) = 7.9, range = 17 to 59). Of the 65 subjects, 35 (53.8%) received buprenorphine and 30 (46.2%) received bupropion. The mean ages were 32.8 years (SD = 7.56, range = 22 to 59) for the buprenorphine group and 32.21 years (SD = 8.45, range = 17 to 51) for the bupropion group (*t* test = −0.205, degrees of freedom (df) = 1, *P* = 0.838). The mean durations of education were 9.12 years (SD = 2.84) for the buprenorphine group and 9.97 years (SD = 3.11) for the bupropion group (*t* test = −1.118, df = 1, *P* = 0.268). The mean durations of methamphetamine dependency were 9.69 years (SD = 6.28) for the buprenorphine group and 7.48 years (SD = 4.65) for the bupropion group (*t* test = 1.523, df = 1, *P* = 0.133).

There were no statistically significant differences between the two groups in regard to age, education, and duration of methamphetamine dependency.

Table 1 indicates *t* tests and ANOVA with repeated measures for craving scores of both groups during 14 days of treatment. As we observe in the buprenorphine group, there are significant differences in craving scores from days 1 to 14 (*P* <0.001). Furthermore, in the bupropion group, there are significant differences in craving scores from days 1 to 14 (*P* <0.001).

**Table 1:** Demographic characteristics of the patients

		Buprenorphine	Bupropion	Statistic	*P* value
Age	Years	32.8 ± 7.56	33.21 ± 8.45	−0.205	0.838
Marital status	Single	14 (25.5%)	10 (18.2%)	2.821	0.244
	Married	14 (25.5%)	14 (25.5%)		
	Divorce	3 (5.5%)	0 (0%)		
Education	Years	9.12 ± 2.84	9.97 ± 3.11	−1.118	0.268
Job	Unemployed	2 (3. 3%)	6 (9.8%)	5.526	0.101
	Employee	5 (8.2%)	1 (1.6%)		
	Self-employed	25 (41%)	21 (34.4%)		
	Student	0 (0%)	1 (1.6%)		
Monthly salary, tomans	<500/000	2 (3.3%)	3 (5%)	0.754	0.385
	500/000–2/000/000	33 (55%)	22 (36.6%)		
Duration of amphetamine addiction	Years	9.69 ± 6.28	7.48 ± 4.65	1.523	0.133

In the mean of craving between the buprenorphine and bupropion groups, there is a significant difference (F = 6.811, df = 13, power = 0.729, *P* = 0.011) (Table 2).

**Table 2:** Assessment of withdrawal craving (with repeated measures) in 14 phases (days)

Group	*N* = 35 Buprenorphine Mean ± SD	*N* = 30 Bupropion Mean ± SD	*t* value	df	*P* value	Power
Day						
Day 1	6.09 ± 2.005	6.6 ± 1.98	−1.04	63	0.303	0.17
Day 2	5.57 ± 1.85	6 ± 1.98	−0. 9	63	0.371	0.14
Day 3	4.71 ± 2.08	5.7 ± 2.28	−1.823	63	0.073	0.43
Day 4	4.29 ± 2.08	4.73 ± 2.35	−0.826	63	0.412	0. 12
Day 5	3.85 ± 2.07	4.63 ± 2.19	−1.466	63	0.148	0. 3
Day 6	3.68 ± 1.97	4.63 ± 2.19	−1.902	63	0.062	0.44
Day 7	2.63 ± 1.78	4. 1 ± 2.56	–	–	0.028*	0.74
Day 8	2.31 ± 1.76	3.97 ± 1.94	−3.599	63	0.001	0.94
Day 9	1.8 ± 1.75	3. 4 ± 2.03	−3.42	63	0.001	0.91
Day 10	1.77 ± 1.93	3.23 ± 2.49	−2.668	63	0.01	0.73
Day 11	1.68 ± 1.73	2.7 ± 2.41	−1.97	63	0.053	0.47
Day 12	1.77 ± 2.16	2.23 ± 2.08	−0.875	63	0.385	0.14
Day 13	0.91 ± 1.59	1.93 ± 2.18	−2.169	63	0.034	0.55
Day 14	0.80 ± 1.29	1.7 ± 2.19	–	–	0.069*	0.45
F	49.074	35.792				
df	13	13				
*P* value	<0.001	<0.001				

*Mann–Whitney test

In the mean of craving between the buprenorphine and bupropion groups, there is a significant difference (F = 6.811, df = 13, power = 0.729, *P* = 0.011). Abbreviations: *df* degrees of freedom, *SD* standard deviation

Overall, a significant main effect of day (*P* <0.001) and group (*P* = 0.011) and a non-significant group-by-day interaction (*P* >0.05) were detected.

All patients had a positive urine drug test for methamphetamine at the beginning of the study. Furthermore, all patients had a negative urine drug test for methamphetamine carried out twice a week during the 14-day interval.

### Adverse effects

Based on our project, all patients were treated in the same way. We monitored all medical conditions, mainly vital signs and also respiratory and cardiovascular signs. All adverse effects of the medications were questioned, monitored, measured, and scored by precise interview three times a day. Only seven patients (two from the bupropion group and five from the buprenorphine group) had significant nausea, vomiting, or hypotension, which were treated by symptom therapy (anti-emetic medications or hydration). This nausea, vomiting, or hypotension was thought to be due to exceeding the patients’ level of buprenorphine or bupropion tolerance. No other severe gastrointestinal, cardiovascular, respiratory, or adverse effects or drug intolerance were observed or reported in the other patients.

Since there was a concern that such drugs could affect hepatic enzymes, blood tests for liver functioning were carried out at baseline and also at follow-ups. Outcome did not illustrate any significant hepatic enzyme changes.

## Discussion

Opioid receptor, mainly mu-opioid receptor, a member of the opioid neuromodulatory system and of the large family of G protein–coupled receptors, is the prominent pharmacological target for the treatment of moderate to severe pain and is of therapeutic value for the management of abuse of amphetamines, opioids, cannabis, alcohol, and other drugs [21–33].

The mechanism of action by which opioids such as buprenorphine prevent methamphetamine craving and dependence is not fully understood; however, there are basic and essential interactions between dopamine and the endogenous opioid neuropeptide systems. Naltrexone (an opioid antagonist) decreases and interrupts the interactions between dopamine and the endogenous opioid neuropeptide systems [19–21]. We theorized that opioid medications such as buprenorphine could enhance the interactions between dopamine and the endogenous opioid neuropeptide systems.

The results of this study are supportive of the effect of buprenorphine and bupropion for the treatment of methamphetamine craving. There was superiority of buprenorphine compared with bupropion. We recommend these two medications as short-term and in-patient treatments to enhance retention or even as longer-term maintenance treatment to reduce relapse.

### Limitations of the clinical trial

Although we had no control group, the fact that the two medications differed significantly in reduction of methamphetamine craving can compensate for this limitation; in the mean of craving between the buprenorphine and bupropion groups, there is a significant difference (f = 6.811, df = 13, power = 0.729, *P* = 0.011).

We require a follow-up to observe what happens when subjects are discharged from a controlled environment. It would be required to specify whether the drugs (buprenorphine/bupropion) prevent short- or long-term relapse.

## Conclusions

It can be considered that buprenorphine is a practical and safe medication for the cessation or reduction of methamphetamine withdrawal craving and is better than bupropion. Use of buprenorphine could be considered for the treatment of methamphetamine craving.

## Data Availability

Please contact the author for data requests. The article does not contain any individual person’s data in any form.

## Abbreviations

ANOVA: Analysis of variance
df: Degrees of freedom
DSM: *Diagnostic and Statistical Manual of Mental Disorders*
SD: Standard deviation
TLC: Thin layer chromatography
VTA: Ventral tegmental area
